# Biochemical characteristics and potential application of a thermostable starch branching enzyme from *Bacillus licheniformis*

**DOI:** 10.1186/s13568-023-01511-4

**Published:** 2023-01-20

**Authors:** Ting Yang, Qianyu Hu, Yu Liu, Rui Xu, Dongrui Wang, Zhongyi Chang, Mingfei Jin, Jing Huang

**Affiliations:** School of Life Sciences, East China Normal University, Shanghai, 200241 China

**Keywords:** 1,4-α-glucan branching enzyme, Slowly digestible starch, Thermostable, *Bacillus licheniformis*

## Abstract

Slowly digestible starch (SDS) has attracted increasing attention for its function of preventing metabolic diseases. Based on transglycosylation, starch branching enzymes (1,4-α-glucan branching enzymes, GBEs, EC 2.4.1.18) can be used to regulate the digestibility of starch. In this study, a GBE gene from *Bacillus licheniformis* (bl-GBE) was cloned, expressed, purified, and characterized. Sequence analysis and structural modeling showed that bl-GBE belong to the glycoside hydrolase 13 (GH13) family, with which its active site residues were conserved. The bl-GBE was highly active at 80 °C and a pH range of 7.5–9.0, and retained 90% of enzyme activity at 70 °C for 16 h. bl-GBE also showed high substrate specificity (80.88 U/mg) on potato starch. The stability and the changes of the secondary structure of bl-GBE at different temperature were determined by circular dichroism (CD) spectroscopy. The CD data showed a loss of 20% of the enzyme activity at high temperatures (80 °C), due to the decreased content of the α -helix in the secondary structure. Furthermore, potato starch treated with bl-GBE (300 U/g starch) showed remarkable increase in stability, solubility, and significant reduction viscosity. Meanwhile, the slowly digestible starch content of bl-GBE modified potato starch increased by 53.03% compared with native potato starch. Our results demonstrated the potential applications of thermophilic bl-GBE in food industries.

**Supplementary Information:**

The online version contains supplementary material available at 10.1186/s13568-023-01511-4.

## Introduction

Nowadays, native starch cannot meet the new requirements of modern industry due to its inherent characteristics, including insoluble in cold water, easy retrogradation, high viscosity, sensitivity to digestion in vivo, etc. (Zeeman et al. 2010). According to the rate and degree of digestibility, starch is categorized into rapidly digestible starch (RDS), slowly digestible starch (SDS) and resistant starch (RS) (Englyst et al. 1992). Rapid digestion and absorption of RDS is prone to induce the generation of metabolic-related diseases, while SDS could slowly absorb and sustained glucose release, and could be used as a new low Glycemic Index (GI) modified starch (Li et al. 2014a; Ludwig 2002; Miao et al. 2015). Consuming low GI foods is beneficial to health, including reducing the risk of chronic metabolic diseases, such as diabetes, obesity and cardiovascular diseases (Goff et al. 2013). However, the content of SDS in food processing was relatively low. To improve the food quality by increasing the content of SDS has become a hot topic in the academic and industrial research. There are many factors that affect the formation of SDS, such as granular structure, crystallinity, the ratio of amylose to amylopectin and the fine structure (Li et al. 2014a, 2018). The previous study reported that α-1,6 glycosidic bonds were not easily digested and absorbed by human digestive enzymes (Gilbert et al. 2013; Kittisuban et al. 2014; Lee et al. 2013a).

Starch branching enzymes (1,4-α-glucan branching enzymes, GBEs, EC 2.4.1.18) break the α- 1,4 glycosidic bond and transfer to the α- 1,6 glycosidic bond (Takata et al. 1994). In the Carbohydrate-Active enZymes database (CAZy) (Henrissat and Bairoch 1996; Janecek et al. 2014; van der Maarel et al. 2002), GBEs are classified into two types: GBEs with (β/α)_8_- barrel structure belonged to glycoside hydrolase family 13 (GH13), and GBEs with (β/α)_7_- barrel structure belonged to glycoside hydrolase family 57 (GH57) (Pal et al. 2010a; Palomo et al. 2011; Santos et al. 2011).It has been reported that the GH13 GBEs have a higher degree of branching effect than that of the GH57 GBEs modified products (Na et al. 2017; Zhang et al. 2019). Therefore, SDS was prepared primarily using GH13 GBE in food industry. Based on the transglycosylation of GBE, the content of α-1,6-glycosidic bonds and the proportion of short branched chains of starch increased, and the average chain length decreased (Le et al. 2009; Lee et al. 2013b). These structural changes can improve the stability, retrogradation resistance and digestion resistance of starch, and can be used to prepare SDS (Hong et al. 2022; Xia et al. 2021). Li et al. found that the SDS content of corn starch modified by GBE from *Rodothermus obamensis* STB05 was increased to 23.90%, and its retrogradation resistance was obviously enhanced (Li et al. 2016, 2014b). Jo et al. found that the digestion rate of sweet potato starch was decreased after treatment with GBE from *Streptococcus* mutans (Jo et al. 2016b). In addition, various applications of starch modified by GBEs have been reported, including paper coating, sport drinking ingredient, spray, and bio-friendly adhesives (Backer and Saniez 2003; Van der Maarel et al. 2014).

However, starch processing typically requires temperatures as high as 70–100 °C (Peng et al. 2021). The high gelatinization temperature of starch indicates that using thermophilic GBEs is an economical and effective strategy for industrial application, which can save a lot of cooling time and prevent microbial contamination, speed up the reaction process and shorten the reaction time (Ban et al. 2020a; Xiao et al. 2020). GBEs have been identified in various organisms (Ball and Morell 2003), such as microorganisms (Chengyao et al. 2021; Suzuki and Suzuki 2016), plants (Sawada et al. 2018; Zhou et al. 2020), and animals (Huynh et al. 2019). However, only a few of these GBEs showed activity at high temperature. Thermophilic GBE from *Bacillus stearothermophilus* displays optimal activity at 55 °C and retained only about 20% activity when incubated at 70 °C for 0.5 h (Ban et al. 2016). *R. obamensis* STB05 GBE remains stable at 70 °C and its optimum temperature is 65 °C (Wang et al. 2019). *Aquifex aeolicus* GBE is stable at 70 °C and displays optimal activity at 75 °C (Van der Maarel et al. 2003). The GBE from *Geobacillus thermoglucosidans* displays optimal activity at 60 °C and lost activity after 20 min of incubation at 70 °C (Ban et al. 2023). The modification process of starch requires GBEs with high catalytic efficiency and high thermal stability (Ban et al. 2018). Higher temperatures are required in industrial applications. Therefore, to improve the thermal stability of *G. thermoglucosidans* GBE, researchers have used several strategies, including introducing salt bridge (Ban et al. 2020c) disulfide bonds (Ban et al. 2020b) and C- terminal truncation (Li et al. 2020a).

However, these mutants do not meet the temperature for starch processing. In this study, a thermophilic GBE from *Bacillus licheniformis* ATCC14580 (bl-GBE) was identified. The bl-GBE was cloned and expressed in *E. coli*, and its biological characteristics were characterized. The bl-GBE’s thermostability was evaluated using the circular dichroism. Then we used the recombinant bl-GBE to treat potato starch to slow down the rate of digestion and improve the solubility and stability.

## Materials and methods

### Chemicals, strains, and plasmids

Additional file 1: Table S1 shows the strains and plasmids used in this study. The *gbe* gene originated from *B. licheniformis* ATCC14580 (GenBank accession no. WP_061576929). All stains were cultured in Luria–Bertani medium (10 g/L tryptone, 5 g/L yeast extract, and 10 g/L NaCl). Isopropyl-β-D-thiogalactopyranoside (IPTG) and ampicillin were provided by Sangon Biotech Co. Ltd. (Shanghai, China). The primer synthesis and sequencing were performed by GENEWIZ (Suzhou, China). Others are commercially available analytical grade chemicals.

### Gene sequence analysis and structure modeling

The standard for determining thermophilic GBE genes was first established based on the physicochemical characteristics of homologous proteins. Therefore, the commercial thermophilic GBE from the *B. stearothermophilus* (Ban et al. 2016) was chosen as a standard protein. The GBE gene in the genome of *B. licheniformis* is located by BLASTP results, and this protein remains uncharacterized. Then, the related literature was searched in the PubMed database using "*Bacillus licheniformis* and thermostable" as the keywords, and the results showed that there were many articles reporting that proteins from *B. licheniformis* had high thermal stability, including thermostable phytase (Zhang et al. 2020), α-amylase (Fincan et al. 2021), cellulase (Yang et al. 2021), etc. It suggested that GBE of *B. licheniformis* (bl-GBE) might have strong thermal stability potential.

The bl-GBE sequence analyses were carried out by the T-COFFEE (http://www.tcoffee.org). Multiple alignments were done using ESPript 3.0, including four characterized thermophilic GBEs and six characterized mesophilic GBEs. The UniProt Accession for these different sources of GBE is shown in Additional file 1: Table S2. The conserved domains and conserved bases were predicted through the website (http://www.ncbi.nlm.nih.gov/Structure/cdd/cdd.shtmln). The 3D structural model of bl-GBE was obtained from AlphaFold Protein Structure Database (https://alphafold.ebi.ac.uk/). The resulting structure was visualized by the PyMOL program. EXPASY PROSITE (https://web.expasy.org/compute_pi/) analyzes the amino acid composition analysis of bl-GBE.

### Expression and purification of recombinant bl-GBE

The gene encoding bl-GBE from *Bacillus licheniformis* ATCC14580 genomic DNA was amplified with the following F1: CGCCATATG ATGGCTGGTGTGAGTGCCTCG (underlined for *Nde* I restriction site) and R1 CCGCTCGAGTCCCTTTTTCGCTCCTCTCT (underlined for *Xho* I restriction site). The bl-GBE was labeled with a histidine tag at the C- terminal. The amplification product and pET32a ( +) were double-cleaved with *Nde* I and *Xho* I. Recombinant plasmid pET32a ( +)/gbe was used to transform competent *Escherichia coli* BL21 (DE3). The recombinant strain was fermented in an LB medium containing 100 ug/mL ampicillin and 0.1 mM IPTG to produce bl-GBE at 20 °C. The strain was harvested by centrifugation and resuspended in wash buffer (20 mM tris, 50 mM NaCl, 5 mM EDTA, 1% Triton X-100, pH 7.5). The cells were then sonicated on ice for 30 min and the cells lysate was collected by centrifugation at 15,000 × *g* for 20 min. Then precipitations were dissolved with 10 mL denaturing buffer (50 mM tris, 6 M guanidine hydrochloride, 10 mM DTT, pH8.0). During the dialysis renaturation process, we dropped the supernatant of denatured protein into renaturation buffer (50 mM PBS, 240 mM NaCl, 10 mM KCl, 0.5 M Arginine, 1 mM EDTA, 0.05% PEG4000, 0.05% Triton X-100, 1 mM GSH, 0.1 mM GSSG; pH 6.5.) at 4 °C for 24 h to form the crude enzyme solution, and then put the solution into the dialysis buffer (20 mM PBS pH6.5) at 4 °C to allow the crude enzyme solution to get the correctly folded protein. Finally, the correctly folded protein is subjected to Ni^2+^ affinity chromatography to obtain the purified protein. The BCA protein assay kit was used to quantify the total protein of cells lysate according to the manufacturer’s instructions. The proportion of target bands to total protein was determined by ImageJ software to calculate the yield of bl-GBE.

### Activity assay of recombinant bl-GBE

According to iodine staining method described by Takata et al., the activity of bl-GBE was assayed (Takata et al. 1994). The enzyme sample (50 µL) was mixed with starch solution (50 uL) consisting of 0.5% amylose (Sigma-Aldrich) and reacted for 30 min and terminated the reaction by adding 2 mL of iodine reagent. The iodine reagent was prepared by adding 0.5 mL of stock solution (2.6 g of I_2_ and 26 g of KI in 100 mL of ddH_2_O) and 0.5 mL 1 M HCl to 129 mL ddH_2_O. One unit (U) of bl-GBE activity was defined as the amount of enzyme that can decrease the absorbance at 660 nm of the amylose-iodine complex by 1% per minute.

### Circular dichroism analysis

The protein secondary structure of bl-GBE was analyzed by circular dichroism (CD) (Applied Photophysics, Surrey, UK). The purified bl-GBE was incubated at 70 °C and 80 °C for 30 min, and the control group was incubated for 30 min at 25 °C. The enzyme concentrations were 0.1 mg/mL dissolved in 10 mM phosphate buffer at pH 8.0, at room temperature. The spectrum from 190 to 260 nm was determined after incubation at 70 °C or 80 °C at room temperature. Protein secondary structural was determine using Circular Dichroism and analyze by Neural Networks (CDNN) (Ioannou et al. 2015; Lighezan et al. 2016).

### Biochemical properties of recombinant bl-GBE

To determine the optimal temperature for bl-GBE activity, purified bl-GBE was tested in a temperature range of 30 °C–100°C. The highest enzyme activity for each reaction condition was defined as 100% activity. The residual activities were then determined by measuring the remaining enzyme activity after incubating the bl-GBE at 70 °C–90 °C for several time intervals. The optimum pH of enzyme activity was measured by incubating enzyme solution at pH values ranging from 4 to 12. The effect of pH on bl-GBE stability was studied by incubating the enzyme at different pH values (pH 4.0–12.0). The residual enzyme activity was then measured. The purified bl-GBE was mixed with metal ion chloride salts (Fe^3+^, Zn^2+^, Cu^2+^, Ca^2+^, Mg^2+^, Mn^2+^, Zn^2+^, Li^+^, Na^+^, Li^+^, and K^+^) and EDTA at concentrations of 1 mM, 5 mM and 10 mM. The residual enzyme activity of bl-GBE was measured. All reactions were repeated in triplicate, and the experimental data displayed was mean ± standard deviation in this work.

### Substrate specificity for recombinant bl-GBE

The substrate specificity of bl-GBE was studied using six starches as substrates, including amylose (Sigma-Aldrich), amylopectin (Sigma-Aldrich), corn starch, wheat starch, soluble starch, and potato starch (Yuanye, Shanghai, China). 0.5% substrate (Ye et al. 2021) solutions were prepared with Tris–HCl buffer (pH8.5, 50 mM), and the enzyme activity determination method is shown above.

### Preparation of bl-GBE modified potato starch

The potato starch (10%, v/w) was gelatinized in a water bath for 30 min, and the bl-GBE (300 U/g starch) was added to begin the reaction at 70 °C for 12 h. Next, bl-GBE in the solution was inactivated by boiling for 20 min. The starch solution added three times the ethanol volume to the precipitated sample by centrifugation at 12,000×*g* for 10 min, after which it was washed with 75% ethanol. The modification potato starch was freeze-dried and pulverized into a powder by a 100-mesh sieve. Samples without bl-GBE addition was used as a control.

### Iodine binding capability analysis

Iodine colorimetric analysis was performed as described (Wickramasinghe et al. 2009). The absorption spectrum and the maximum absorption wavelength (λ_max_) were analyzed by scanning wavelengths from 300 to 800 nm.

### Rheological properties analysis

According to a previous method (Guo et al. 2016), the steady-state and dynamic rheology of 5% (w/v) native and bl-GBE treated starch was measured at 25 °C using a MARSIII Rotational Rheometer. A program using a parallel plate with a diameter of 40 mm was selected as a steady-state test. After setting a gap of 1 mm, the excess starch solution was scraped off, and the apparent viscosity (η) of the starch paste was measured as the shear rate increased.

### Solubility and dissolution stability analysis

Previously reported protocols were used to measure the solubility of the starch (Keeratiburana et al. 2020). The 0.5 g sample was added to 25 mL of distilled water and vibrated for 15 min. The sample solution was heated in boiling water for a few minutes and then centrifuged at 6000×*g* for 15 min. The supernatant was then dried to a constant weight at 110 °C. The starch weight was calculated. The starch was configured to 10% solution, and the stability of the solution was observed at different times (0 h, 24 h) at 4 °C.

$$ {\text{Solubility }}\left ( \% \right) = \left ( {{\text{weight of soluble starch}}/{\text{weight of starch sample}}} \right)*{1}00. $$

### In vitro digestion properties analysis

Based on the method established by Englyst (Englyst et al. 1992), the method of in vitro digestibility of bl-GBE modified starch is slightly changed as previously reported (Jo et al. 2016a). Porcine pancreatic α -amylase (2 g) (P7545, Sigma Chemical Reagent Co., Ltd) was added to distilled water (24 mL) with stirring for 10 min and centrifuged at 1500×*g* for 10 min. The enzyme supernatant (20 mL) was transferred to a test tube and mixed with amyloglucosidase (0.4 mL) (A7095, Sigma Chemical Reagent Co., Ltd) and distilled water (3.6 mL). The pH of the modified starch sample was adjusted to 5.2, and incubated at 37 °C for 10 min before adding 0.75 mL of mixed enzyme solution. Subsequently, the reaction mixture was aliquoted at 20 min and 120 min and immediately boiled for 30 min to inactivate the enzyme. The content of glucose in the hydrolysate after modified starch digestion was determined by GOD-POD kit (Shin et al. 2010). The content of rapidly digestible starch (RDS), slowly digestible starch (SDS), and resistant starch (RS) is calculated as follows:

$$ \begin{aligned} & {\text{RDS }}\left ( \% \right) = \left ( {{\text{G2}}0 - {\text{G}}0} \right) \, \times \, 0.{9 } \times { 1}00 \\ & {\text{SDS }}\left ( \% \right) = \left ( {{\text{G12}}0 - {\text{G2}}0} \right) \, \times \, 0.{9 } \times { 1}00 \\ & {\text{RS }}\left ( \% \right) = {1}00\% - {\text{RDS }}\left ( \% \right) - {\text{SDS }}\left ( \% \right) \\ \end{aligned} $$

The G0 is the free glucose content, G20 and G120 are defined as the produced glucose after 20 min and 120 min of hydrolysis.

## Results

### Predicted 3D structure of bl-GBE

**Fig. 1:**
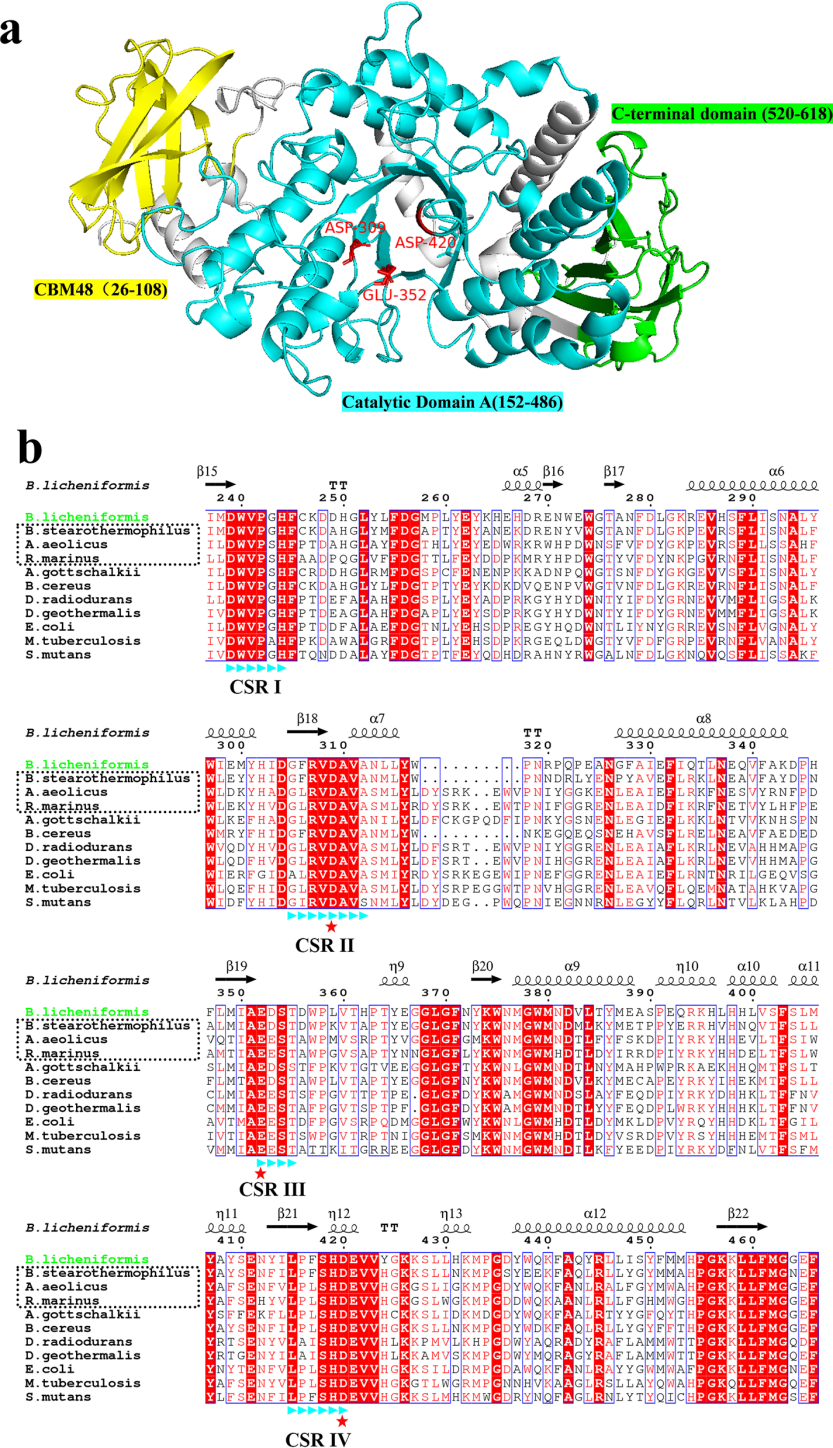
Multiple alignment and homology modeling analysis of bl-GBE **a** 3D structural model of bl-GBE protein was generated using AlphaFold. Glu352 (a nucleophile), Asp309, and Asp420 (acid/base catalyst) are the three catalytic residues conserved in members of the GH13 family. The position of the 3 domains is shown in different colors; yellow: the N-terminal domain, blue: catalytic domain A, and green: the C-terminal domain, **b** Sequence alignment of GBEs. Conserved residues are designated with red squares and white lettering, and similar residues are designated with red lettering. GBE amino acid sequences from different sources are aligned. GBEs in black box has been commercialized. The secondary structure is derived from the predicated structure of bl-GBE (AlphaFold). The highly conserved catalytic residues are indicated with red stars. The four highly conservative motifs (CRS) are in the blue triangle

bl-GBE is an uncharacterized conserved protein annotated as a “1,4-alpha-glucan branching enzyme” in the *B. licheniformis* genome (https://www.ncbi.nlm.nih.gov/protein/WP_061576929.1/). Thus, this study is the first report to characterize bl-GBE. GBE has been reported to consist of domain A, domain C, and a family of carbohydrate-binding modules 48 (CBM48) (Janecek et al. 2019; Suzuki and Suzuki 2016). The 3D structure of bl-GBE was predicated through AlphaFold database. The positions of catalytic amino acids and multiple domains of the bl-GBE are shown in Fig. 1a. CBM48 includes residues 26–108, the catalytic domain contains residues 152–486 and the C-terminal domain contains residues 520–618. Ten characterized thermophilic GBEs (including three commercial GBE) and mesophilic GBEs were selected for multiple sequence alignment with bl-GBE and their thermal stability is shown in Table 5. The most homologous sequence was that of the thermophilic GBE from *B. stearothermophilus* (64.2%). Studies have shown that GBEs have four conserved regions that are present in the α-amylase family and contain all catalytic and substrate-binding residues (Hayashi et al. 2015; Janecek et al. 2014; Takata et al. 1994). Figure 1b showed the alignment sequence of the catalytic domain A region which both thermophilic and mesophilic GBEs showed significant sequence identity. The GBEs sequence contains four conserved regions bl-GBE possesses conserved catalytic triad amino acid sequences at Asp309, Glu352, and Asp420, which is common in GBEs belonging to GH13.

### Heterologous expression and purification of bl-GBE

**Fig. 2:**
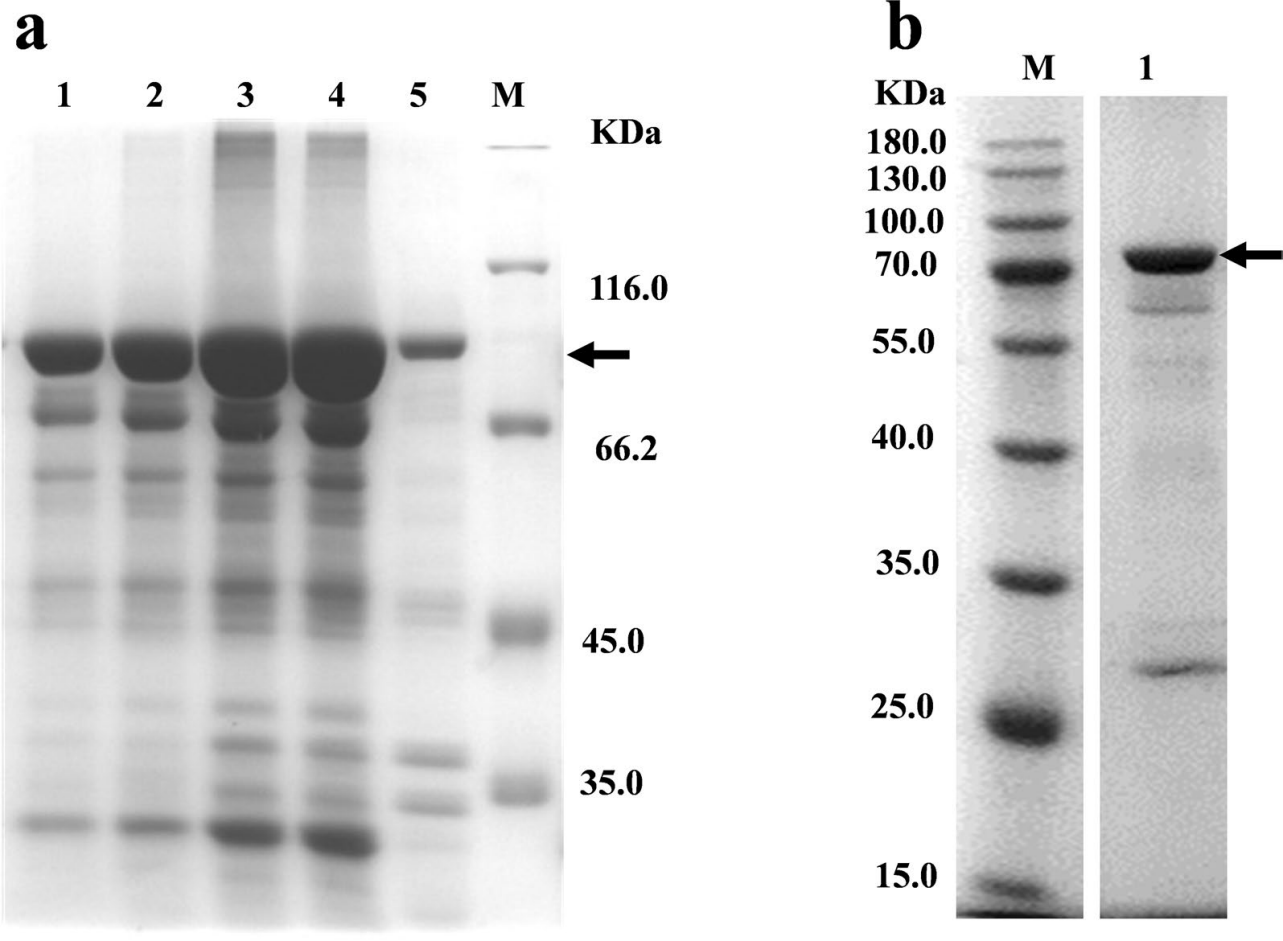
Expression, and purification of recombinant bl-GBE **a** SDS-PAGE analysis of bl-GBE expressed as inclusion bodies in *BL21 (DE3)*. Lane M, Unstained Protein MW Marker; lanes 1–4, samples induced at 20 °C for 12, 24, 48, and 72 h, respectively; lane 5, uninduced control, **b** SDS-PAGE analysis of the purified recombinant bl-GBE protein. Lane M, unstained Protein MW Marker; lane1: purification sample of recombinant bl-GBE. The black arrow indicates the position of bl-GBE protein band

To verify the biological properties of the candidate bl-GBE, the 1884 bp of the bl-GBE sequence was successfully amplified from the *B. licheniformis* ATCC14580 genome and the DNA sequence was confirmed by sequencing. To produce bl-GBE protein in *E. coli* BL21 (DE3), we used a pET32 (+) expression vector system containing an efficiently T7 promoter. After induction of expression by the recombinant strain, it was found that almost all of the proteins were expressed in inclusion bodies, and the bands shown by SDS-PAGE corresponded to the theoretical bl-GBE molecular mass of 73.8KDa (Fig. 2a). Higher expression levels were observed in fermentation for 72 h at 20 °C, achieving the final yield of about 650 mg/L. Next, the recombinant bl-GBE was subjected to renaturation, denaturation and purification. The position of the purified enzyme on SDS-PAGE was shown in Fig. 2b. The bl-GBE recovered with specific enzyme activity of 77.2 U/mg for amylose and total protein recovery was 23.3% (Additional file 1: Table S3).

### Biochemical properties of recombinant bl-GBE

**Fig. 3:**
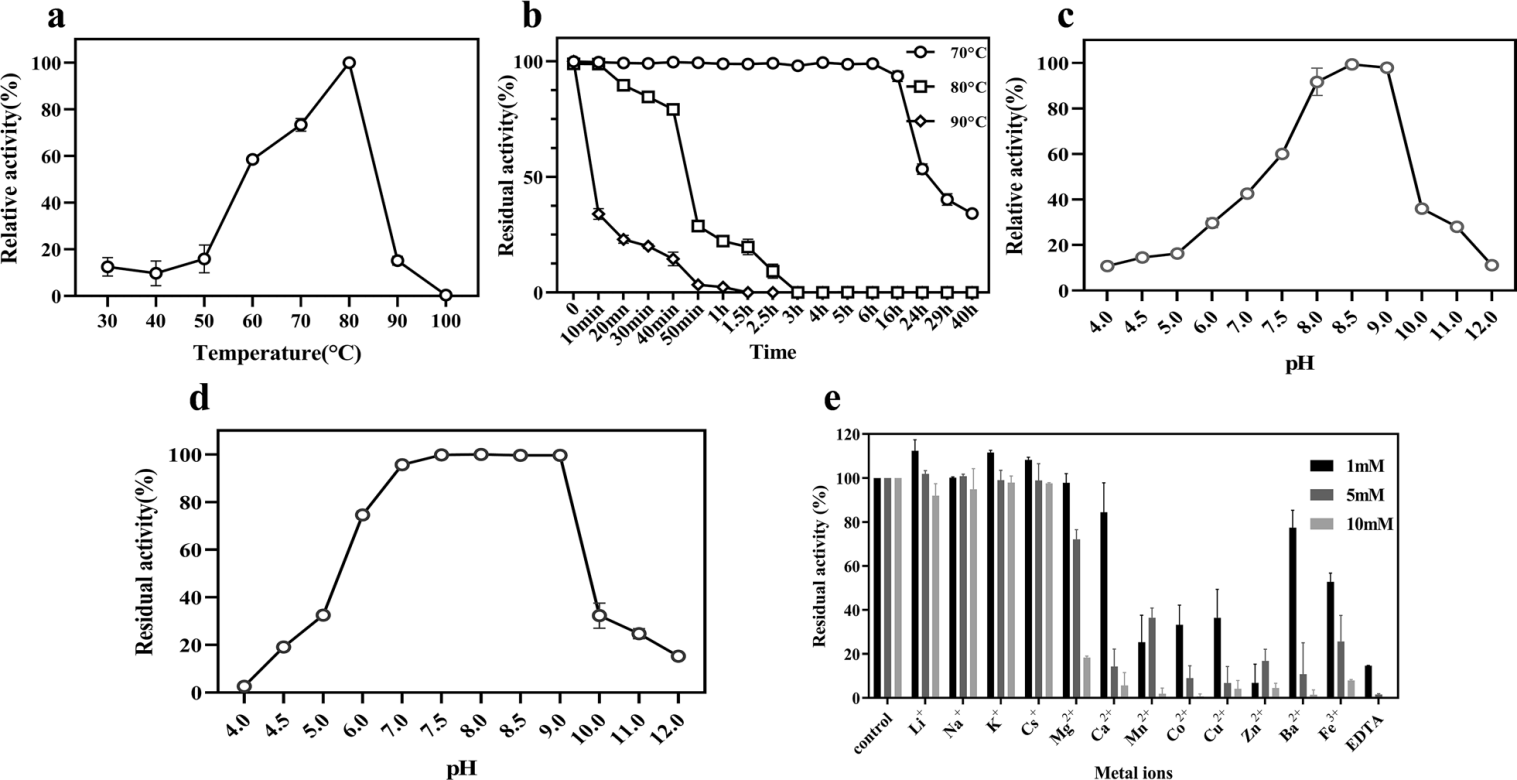
Characterization of bl-GBE. The maximum enzyme activity in each group was set at 100%, **a** bl-GBE activity at different temperatures. Reactions were performed at temperatures ranging from 30 to 100 °C, **b** bl-GBE residual activity after incubation for different durations at 70 °C, 80 °C, and 90 °C, **c** Effect of pH on bl-GBE activity. Activity assays for purified recombinant bl-GBE were performed at 80 °C temperature with pH ranging from 4.0 to 12.0, **d** pH stability of bl-GBE. bl-GBE was preincubated for 12 h at pH ranging from 4.0 to 12.0 before measuring residual activity, **e** Effect of reagents on enzyme activity of bl-GBE

We analyzed the optimum temperature of bl-GBE by conducting a series of enzymatic assays under varying conditions. bl-GBE activity increased from 10 to 100% with an increase in temperature from 30 to 80 °C (Fig. 3a). bl-GBE showed a maximum activity at 80 °C. The bl-GBE was incubated under high-temperature conditions varying from 70 to 90 °C for 10 min to 40 h. The results showed the bl-GBE retained 90% of enzyme activity at 70 °C for 16 h. At 80 °C for 30 min, 80% activity remained (Fig. 3b). bl-GBE lost 80%-85% activity when preincubated at 80 °C for 1–1.5 h and all of its activity when incubated for 3 h or more (Fig. 3b). After incubation at 90 °C for 10 min, the residual activity was only 30%.

The effect of pH on bl-GBE activity was investigated. The results showed the highest activity at pH 8.5 with 50% activity from pH 7.5 to pH 9.0 (Fig. 3c). bl-GBE activity increased from 10 to 60%, with an increase in pH from 4.0 to 7.5. The relative activity of bl-GBE was only 10%–30% at pH 10.0–12.0. The bl-GBE was stable from pH 7.0 to 9.0 after incubation for 12 h and the remaining 75% of its initial activity at pH 6.0 (Fig. 3d). bl-GBE activity rapidly decreased under strongly acidic or strong basic conditions, retaining only 15%-30% of its activity, and lost all activity at pH 4.0.

Additionally, the stability and activity of the enzyme are most important in commercial applications and could be affected by metal ions. Fe^3+^, Ba^2+^, Cu^2+^, Mn^2+^, and Zn^2+^ strongly inhibited enzyme activity, and as the concentration increased, the degree of inhibition was greater. bl-GBE activity had no significant effect on some metal ions, such as Li^+^, Na^+^, Cs^+^, and K^+^ (Fig. 3e). With the presence of chelating agent EDTA, the activity of bl-GBE almost completely lost (Fig. 3e).

### Substrate specificity for recombinant bl-GBE

We evaluated the activity of various substrates for the recombinant bl-GBE. As shown in Table 1, the highest activity of 80.88 U/mg was obtained towards potato starch, while amylose, amylopectin, wheat starch, soluble starch, and corn starch are 95.45%, 59.71%, 75.1%, 86%, and 50.88%, relative to that of potato starch, respectively. Then the enzyme kinetics of bl-GBE were studied.

**Table 1:** Substrate specificity of the bl-GBE

Substrates	Relative activity (%)	Specific activity (U/mg)
Amylose	95.45 ± 1.09	77.20 ± 1.59
Amylopectin	59.71 ± 3.53	48.3 ± 3.51
Potato starch	100 ± 0.15	80.88 ± 3.12
Soluble starch	86.00 ± 2.15	69.56 ± 4.99
Wheat starch	75.1 ± 1.94	60.74 ± 0.19
Corn starch	50.88 ± 2.54	41.16 ± 3.65

### Secondary structure measurements of recombinant bl-GBE

**Fig. 4:**
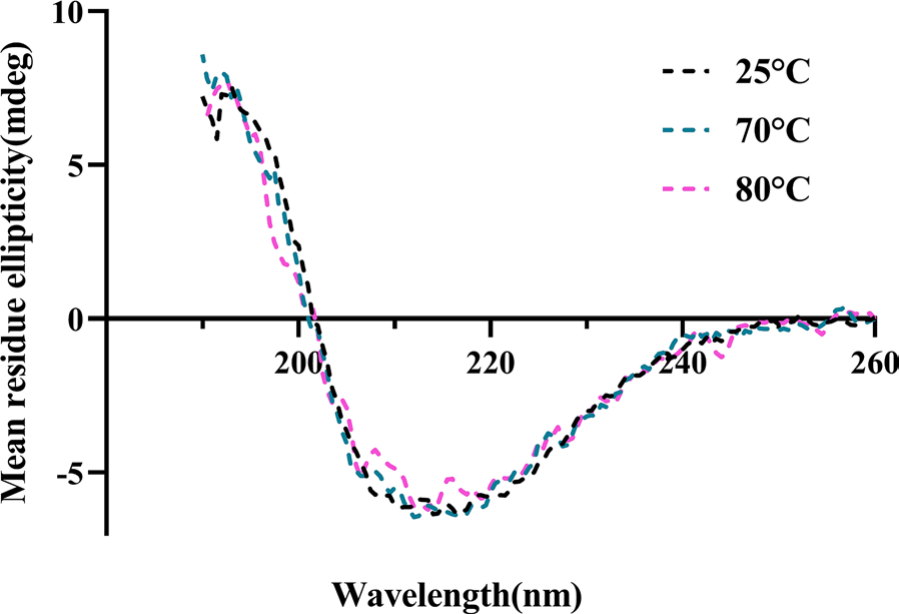
CD spectra of the enzyme after incubation at different temperature conditions

The CD spectrum is used to study the secondary structure of proteins (Horchani et al. 2014; Yang et al. 2008). In this study, the structural changes of the enzyme at high temperature were determined by CD spectra to study the thermal stability of bl-GBE. To observe the secondary structure changes of bl-GBE in detail, three temperatures (25 °C, 70 °C, and 80 °C) were selected. Native bl-GBE had 19.52% α-helix, 34.63% β-fold, 14.01% β-turn, and 31.83% random coil (Table 2). CD data showed no obvious change in the secondary structure of bl-GBE when incubated at 70 °C for 30 min (Fig. 4). After bl-GBE was incubated at 80 °C for 30 min, the β-fold in its secondary structure was increased by 5.98% and the α-helix decreased by 5.17%. These results indicate that high temperature destroyed the bl-GBE 's secondary structure.

**Table 2:** Secondary structural element contents of bl-GBE incubated at different temperatures for 30 min

Structure	Samples		
	25 °C	70 °C	80 °C
α-Helix (%)	19.52	18.98	18.51
β-fold (%)	34.63	35.95	36.78
β-Turn (%)	14.01	13.71	13.39
Radom Coil (%)	31.83	31.35	31.4

The contents were estimated using CDNN

### Iodine binding capability analysis

**Fig. 5:**
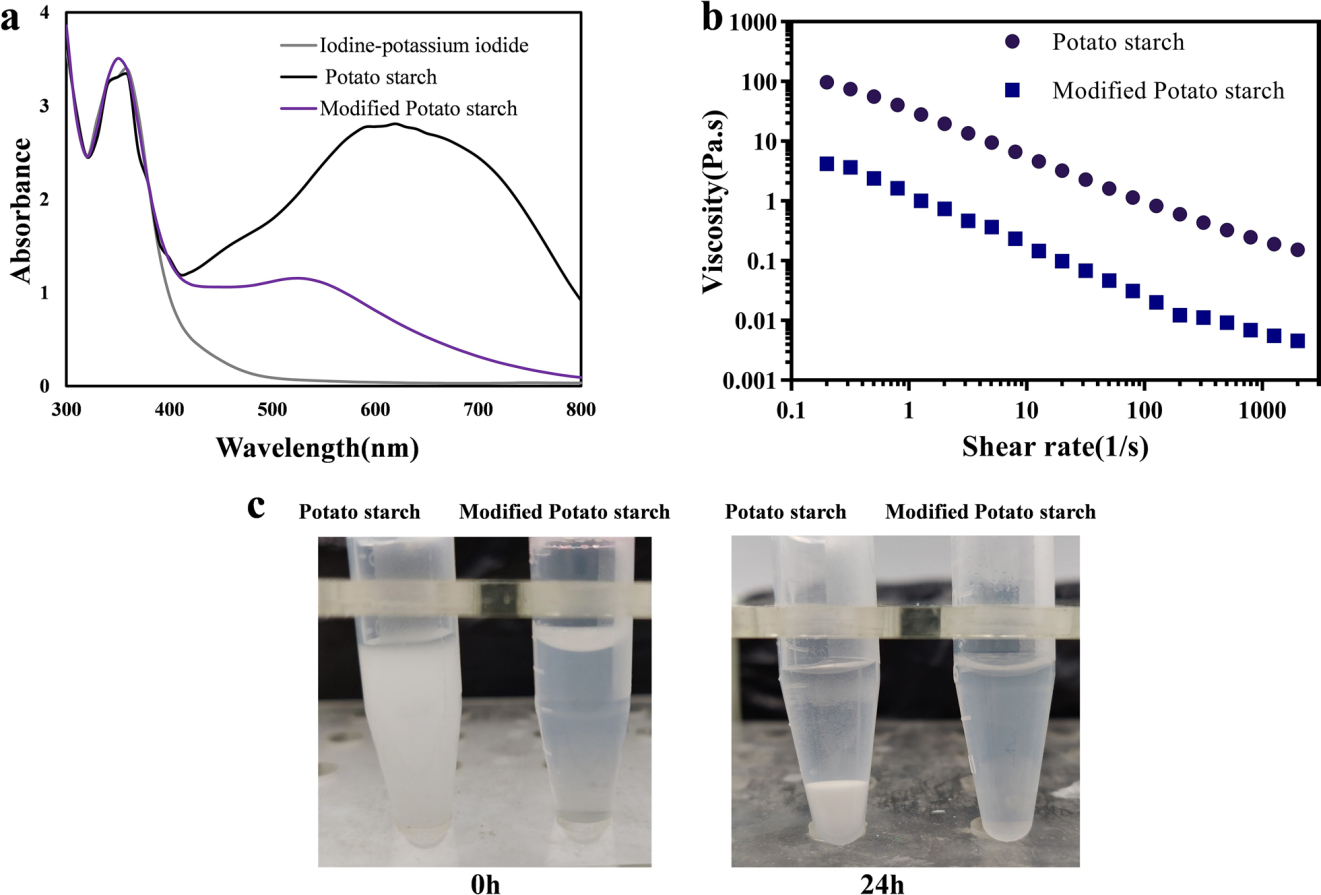
Physicochemical characteristics of native and bl-GBE-modified potato starch **a** Wavelength scanning profile of iodine complex formed by starch, **b** Static rheological properties of bl-GBE modified potato starch, **c** Native and bl-GBE modified potato starch dissolution stability at different times (0 h, 24 h)

Amylose and amylopectin can form a single-chain complex with iodine and the affinity of the starch structure for iodine can be estimated by spectrophotometry (Jiang et al. 2014). The wavelength spectrum of the starch-iodine complex can reflect the structural change of the bl-GBE by a maximum wavelength movement and the decreasing absorption value. The complex formed by potato starch-iodine is dark blue with a maximum absorption wavelength of 620 nm and an absorbance of 2.81 (Fig. 5a). The branching reaction was characterized by a decrease in iodine binding capacity (Xia et al. 2021), which was consistent with our results. The maximum absorption wavelength of the modified starch-iodine complex is at approximately 530 nm, and the absorption value of the complex at approximately 530 nm and is significantly reduced to 1.15 (Fig. 5a). This indicated that bl-GEB increased the α-1,6-branching points of starch.

### Rheological measurement

The complex dynamic viscosity (η*) of starch at 25 °C is shown (Fig. 5b). The results indicated that the apparent viscosity of all the sample pastes decreased as the shear rate increased, and the potato starch solution is a shear-thinning fluid. The viscosity value of the enzyme-modified starches decreased by one order of magnitude of unmodified starch, which indicated that the viscosity of bl-GBE modified starch is lower. Therefore, the modified starch can be applied to in high-concentration food industry with low viscosity.

### Solubility and dissolution stability of bl-GBE modified potato starch

The solubility reflected the degree of combination between starch and water, which was closely related to the internal structure of starch. We found that 10% of potato starch with the aqueous solution of the potato starch was almost insoluble in cold water. The enzyme-modified potato can be quickly dispersed in rapid dissolution. After standing for 24 h, the potato starch was precipitated, as shown in Fig. 5c, while the enzyme-modified starch was still in a uniformly stable solution state. The results of the solubility calculations for the native and bl-GBE treated-potato starches were shown in Table 3. This indicated that bl-GBE modified starch solubility (70.5%) approached 10 times that of native starch (7.9%). These results demonstrated that bl-GBE-modified starch can increase the solubility and stability of starch.

**Table 3:** Solubility of the native and bl-GBE modified potato starch

Sample	Solubility (%)
Control	7.9 ± 0.4
Modified potato starch	70.5 ± 0.5

### In vitro digestibility of bl-GBE modified potato starch

In vitro digestion experiment was carried out to investigate the change of digestibility of starch after denaturation. The content of RDS showed a downward trend. RDS decreased from the 51.28% to 35.18% (Table 4). The content of SDS increased from 25.21 to 38.58%, an increase of 12.37%. RS content increased from 23.71 to 26.39%, an increase of 1.68%.

**Table 4:** The digestibility of potato starch modified with bl-GBE

Samples	RDS content (%)	SDS content (%)	RS content (%)
Control	51.28 ± 0.14	25.21 ± 1.06	22.71 ± 1.16
Modified potato starch	35.18 ± 0.21	38.58 ± 2.06	26.39 ± 0.75

The data were expressed as means and standard deviations (SD), which were calculated from three repetitions of each treatments

## Discussion

To meet the requirements of starch industry, it was necessary to find an ideal GBE with both high activity and thermal stability. Sequence alignment analysis showed that the similarity between bl-GBE and thermophilic GBEs is in the range of 40–65% (Additional file 1: Table S2). These results confirmed catalytic functionality of bl-GBE. bl-GBE activity reached 80.88 U/mg for potato starch after renaturation. The specific activity of bl-GBE was 77.20 U/mg for amylose, which was higher than *G. stearothermophilus* (36 U/mg) (Aga et al. 2010), *Rhizomucor miehei* (10.70 U/mg) (Wu et al. 2014), and *Mycobacterium tuberculosis* (63.75U/mg) (Pal et al. 2010b). This showed that the higher catalytic efficiency of bl-GBE was conducive to its industrial applications. Considering the application of bl-GBE in functional material synthesis and the food industry in the future, we would try to soluble express in food-safe strains.

Temperature markedly affected enzyme activity and influenced molecular structure (Ping et al. 2017). Table 5 showed the optimum temperature and thermal stability of GH13 GBE from bacteria, which have been heterologous expressed and characterized in the BRENDA enzyme database. GBE from *A. aeolicus* has the high optimum temperature (75 °C). However, its specific enzyme activity is lower (4.9U/mg) (Wang et al. 2019), resulting in high production cost (Van der Maarel et al. 2003). Recombinant thermophilic bl-GBE was first characterized in this study and has the highest optimum temperature (80 °C) in the GH13 family. And meets the high temperature conditions required by industry. The GBE from *B. stearothermophilus* lost 84% of its activity in 30 min at 70 °C (Kiel et al. 1991), while bl-GBE still retains 30% of enzyme activity at 70 °C in 40 h. Therefore, the bl-GBE is more stable than GBE from *B. Stearothermophilus.* The bl-GBE has a longer half-time (24 h) at 70 °C, while *A. gottschalkii* lost 50% of its activity at 55 °C for 55 min (Thiemann et al. 2006) which is much higher than that of other GBEs (Ban et al. 2018).These results confirmed that bl-GBE has strong thermal stability and optimum temperatures. Three GBEs in the GH13 family have been commercialized, demonstrating the commercial potential of GBEs. At present, the three commercial *R. obamensis* STB05*, B. Stearothermophilus* and *A.aeolicus* GBEs are respectively used for the production of high-branched functional starch (Roussel et al. 2013a), the synthesis of glycogen (Kajiura et al. 2009), and the preparation of highly-branched cyclic dextrin (Takata et al. 1996). However, their optimum temperature is lower than 80 °C. It indicated that bl-GBE had better commercial prospect in starch modification.

**Table 5:** Optimal temperature and thermal stability of branching enzymes from different organisms. bl-GBE in this study is displayed in bold fonts

Organism	Thermal stability	Optimal temperature	References
*Bacillus subtilis*	–	30 °C	Lee et al. (2008)
*Bacillus cereus*	Retain activity at 50 °C for 30 min	30 °C	Takata et al. (2010)
*Escherichia coli*	Retain 50% activity at 25–37 °C	30 °C	Guan et al. (1997)
*Mycobacterium tuberculosis*	Less than 37 °C	30 °C	Garg et al. (2007)
*Deinococcus geothermalis*	Retain full activity at 60 °C	34 °C	Palomo et al. (2009)
*Deinococcus radiodurans*	Retain full activity at 60 °C	34 °C	Palomo et al. (2009)
*Butyrivibrio fibrisolvens* H17C	–	37 °C	Rumbak et al. 1991)
*Streptococcus mutans*	No more than 40 °C	37 °C	Kim et al. (2008)
*Anaerobranca gottschalkii*	Stable at 50 °C for more than 6 h	50 °C	Thiemann et al. (2006)
*Geobacillus mahadia*	The half-life70°C is 5 h, respectively	55 °C	Mohtar et al. (2016)
*Caldicellulosiruptor bescii*	–	60 °C	Park et al. (2019)
*Bacillus stearothermophilus*	Stable up to 60 °C	55 °C	Takata et al. (1994)
*Rhodothermus obamensis STB05*	Stable up to 80 °C	65 °C	Roussel et al. (2013b)
*Aquifex aeolicus*	Stable up to 90 °C	75 °C	Choi et al. (2009)
Bacillus licheniformis ATCC 14,580	**Stable up to 70 °C for 16 h**	**80** °C	**This study**

The CD spectra found a decrease in α-helix content after bl-GBE incubation at 80 °C for 30 min. According to the thermal stability results of this study, the enzyme lost 20% of its activity after incubation under the same conditions. The results revealed that high temperatures reduced its activity by changing the secondary structure of bl-GBE. The native bl-GBE secondary structure contained well α -helix, while its content decreased with increasing the temperature. The content of β-sheet also changed significantly under similar denaturation conditions. This mechanism of bl-GBE inactivation differs from that reported for *R. obamensis* GBE, in which the contents of α-helix and β-fold were significantly decreased, and the content of random coil was significantly increased, respectively (Wang et al. 2019). However, this is similar to the previously reported enhancement of the thermal stability of *G. thermoglucosidasius* GBE by K^+^ or Na^+^ in the presence of glycerol by increasing the α-helix content (Ban et al. 2017).

In addition, compared with native potato starch, bl-GBE modified potato starch showed increased branch density, solubility, stability, SDS content, and decreased viscosity. Compared with the GBE-treated potato starch from *Aspergillus niger* with the solubility increased to up to 64.6% (Guo et al. 2019), the solubility level of bl-GBE modified potato starch (70%) was relatively high. This might be due to the fact that the short-chain structure of starch was provided with high branch density by enzyme modification of starch, which inhibited the recrystallization of amylose, and resulted in more hydrogen bond exposure, enhancing the hydration properties and solubility of starch. Gelatinized starch has poor flowability and makes it difficult to perform the enzyme-catalyzed reaction, A decrease in viscosity of bl-GBE modified starch could be due to a decrease in the amylose content and entanglement between the amylose chains. In addition, due to the short length and high density of the branched chain, the interaction between molecular chains is weakened, which reduces the content of the junction zone in the gel network structure, resulting in greater shear-thinning power. The more important industrial application of GBEs is the production of SDS. Some studies have reported that differences in starch digestibility can be attributed to a variety of factors (Man et al. 2015; Tester et al. 2004). It has been reported that the reduced digestibility of some GBE-modified starches may be related to the chain length, branch density and molecular weight of the starch (Guo et al. 2019; Li et al. 2020b). Compared with native potato starch, the SDS content of bl-GBE modified potato starch was increased by 53.03% and this is higher than the SDS content of GBE from *Aquabacterium* sp. strain A7-Y modified starch (Chengyao et al. 2021). Based on the potential application of bl-GBE, we will continue to analyze the properties of bl-GBE modified starch to improve the industrial application value of highly branched starch.

## Supplementary Information

**Additional file 1: Table S1.** Strains and plasmids used in this study.** Table S2.** The identity percentage for alignment between bl-GBE and ten GBE sequences obtained from literature mining.** Table S3.** Relevant index of protein purification.

## Data Availability

The datasets supporting the conclusions of this article are included within the article and its Additional file 1.

## References

[CR1] Aga H, Okamoto I, Taniguchi M, Kawashima A, Abe H, Chaen H, Fukuda S (2010). Improved yields of cyclic nigerosylnigerose from starch by pretreatment with a thermostable branching enzyme. J Biosci Bioeng.

[CR2] Backer D, Saniez M-H (2003) Soluble highly branched glucose polymers prepared by enzymic modification of starch or starch derivatives. EP1369432A2

[CR3] Ball SG, Morell MK (2003). From bacterial glycogen to starch: understanding the biogenesis of the plant starch granule. Annu Rev Plant Biol.

[CR4] Ban X, Dhoble AS, Li C, Gu Z, Hong Y, Cheng L, Holler TP, Kaustubh B, Li Z (2020). Bacterial 1,4-alpha-glucan branching enzymes: characteristics, preparation and commercial applications. Crit Rev Biotechnol.

[CR5] Ban X, Dhoble AS, Li C, Zhang Y, Gu Z, Cheng L, Hong Y, Li Z (2017). Potassium and sodium ions enhance the activity and thermostability of 1,4-alpha-glucan branching enzyme from *Geobacillus thermoglucosidasius* in the presence of glycerol. Int J Biol Macromol.

[CR6] Ban X, Li C, Gu Z, Bao C, Qiu Y, Hong Y, Cheng L, Li Z (2016). Expression and biochemical characterization of a thermostable branching enzyme from *Geobacillus thermoglucosidans*. J Mol Microbiol Biotechnol.

[CR7] Ban X, Li C, Zhang Y, Gu Z, Cheng L, Hong Y, Li Z (2020). Importance of C-terminal extension in thermophilic 1,4-alpha-glucan branching enzyme from *Geobacillus thermoglucosidans* STB02. Appl Biochem Biotechnol.

[CR8] Ban X, Liu Y, Zhang Y, Gu Z, Li C, Cheng L, Hong Y, Dhoble AS, Li Z (2018). Thermostabilization of a thermophilic 1,4-alpha-glucan branching enzyme through C-terminal truncation. Int J Biol Macromol.

[CR9] Ban X, Wang T, Fan W, Li C, Gu Z, Cheng L, Hong Y, Li Z (2023). Thermostability and catalytic ability enhancements of 1,4-alpha-glucan branching enzyme by introducing salt bridges at flexible amino acid sites. Int J Biol Macromol.

[CR10] Ban X, Wu J, Kaustubh B, Lahiri P, Dhoble AS, Gu Z, Li C, Cheng L, Hong Y, Tong Y, Li Z (2020). Additional salt bridges improve the thermostability of 1,4-alpha-glucan branching enzyme. Food Chem.

[CR11] Chengyao X, Yan Q, Chaonan D, Xiaopei C, Yanxin W, Ding L, Xianfeng Y, Jian H, Yan H, Zhongli C, Zhoukun L (2021). Enzymatic properties of an efficient glucan branching enzyme and its potential application in starch modification. Protein Expr Purif.

[CR12] Choi SS, Danielewska-Nikiel B, Kojima I, Takata H (2009). Safety evaluation of 1,4-alpha-glucan branching enzymes from *Bacillus stearothermophilus* and *Aquifex aeolicu*s expressed in *Bacillus subtilis*. Food Chem Toxicol.

[CR13] Englyst HN, Kingman SM, Cummings JH (1992). Classification and measurement of nutritionally important starch fractions. Eur J Clin Nutr.

[CR14] Fincan SA, Ozdemir S, Karakaya A, Enez B, Mustafov SD, Ulutas MS, Sen F (2021). Purification and characterization of thermostable alpha-amylase produced from *Bacillus licheniformis* So-B3 and its potential in hydrolyzing raw starch. Life Sci.

[CR15] Garg SK, Alam MS, Kishan KV, Agrawal P (2007). Expression and characterization of alpha- (1,4)-glucan branching enzyme Rv1326c of *Mycobacterium tuberculosis* H37Rv. Protein Expr Purif.

[CR16] Gilbert RG, Wu AC, Sullivan MA, Sumarriva GE, Ersch N, Hasjim J (2013). Improving human health through understanding the complex structure of glucose polymers. Anal Bioanal Chem.

[CR17] Goff LM, Cowland DE, Hooper L, Frost GS (2013). Low glycaemic index diets and blood lipids: A systematic review and meta-analysis of randomised controlled trials. Nutr Metab Cardiovas.

[CR18] Guan H, Li P, Imparl-Radosevich J, Preiss J, Keeling P (1997). Comparing the properties of *Escherichia coli* branching enzyme and maize branching enzyme. Arch Biochem Biophys.

[CR19] Guo L, Deng Y, Lu L, Zou F, Cui B (2019). Synergistic effects of branching enzyme and transglucosidase on the modification of potato starch granules. Int J Biol Macromol.

[CR20] Guo L, Hu J, Zhang J, Du X (2016). The role of entanglement concentration on the hydrodynamic properties of potato and sweet potato starches. Int J Biol Macromol.

[CR21] Hayashi M, Suzuki R, Colleoni C, Ball SG, Fujita N, Suzuki E (2015). Crystallization and crystallographic analysis of branching enzymes from *Cyanothece* sp ATCC 51142. Acta Crystallogr F Struct Biol Commun.

[CR22] Henrissat B, Bairoch A (1996). Updating the sequence-based classification of glycosyl hydrolases. Biochem J.

[CR23] Hong MG, Yoo SH, Lee BH (2022). Effect of highly branched alpha-glucans synthesized by dual glycosyltransferases on the glucose release rate. Carbohydr Polym.

[CR24] Horchani H, Bussieres S, Cantin L, Lhor M, Laliberte-Gemme JS, Breton R (2014). Enzymatic activity of Lecithin:retinol acyltransferase: a thermostable and highly active enzyme with a likely mode of interfacial activation. Biochim Biophys Acta.

[CR25] Huynh N, Ou Q, Cox P, Lill R, King-Jones K (2019). Glycogen branching enzyme controls cellular iron homeostasis via Iron Regulatory Protein 1 and mitoNEET. Nat Commun.

[CR26] Ioannou JC, Donald AM, Tromp RH (2015). Characterising the secondary structure changes occurring in high density systems of BLG dissolved in aqueous pH 3 buffer. Food Hydrocolloid.

[CR27] Janecek S, Marecek F, MacGregor EA, Svensson B (2019). Starch-binding domains as CBM families-history, occurrence, structure, function and evolution. Biotechnol Adv.

[CR28] Janecek S, Svensson B, MacGregor EA (2014). alpha-Amylase: an enzyme specificity found in various families of glycoside hydrolases. Cell Mol Life Sci.

[CR29] Jiang H, Miao M, Ye F, Jiang B, Zhang T (2014). Enzymatic modification of corn starch with 4-alpha-glucanotransferase results in increasing slow digestible and resistant starch. Int J Biol Macromol.

[CR30] Jo AR, Kim HR, Choi SJ, Lee JS, Chung MN, Han SK, Park CS, Moon TW (2016). Preparation of slowly digestible sweet potato Daeyumi starch by dual enzyme modification. Carbohydr Polym.

[CR31] Jo AR, Kim HR, Choi SJ, Lee JS, Chung MN, Han SK, Park CS, Moon TW (2016). Preparation of slowly digestible sweet potato Daeyumi starch by dual enzyme modification. Carbohyd Polym.

[CR32] Kajiura H, Kakutani R, Akiyama T, Takata H, Kuriki T (2009). A novel enzymatic process for glycogen production. Biocatal Biotransform.

[CR33] Keeratiburana T, Hansen AR, Soontaranon S, Blennow A, Tongta S (2020). Pre-treatment of granular rice starch to enhance branching enzyme catalysis. Carbohydr Polym.

[CR34] Kiel JA, Boels JM, Beldman G, Venema G (1991). Molecular cloning and nucleotide sequence of the glycogen branching enzyme gene (glgB) from *Bacillus stearothermophilus* and expression in *Escherichia coli* and *Bacillus subtilis*. Mol Gen Genet.

[CR35] Kim EJ, Ryu SI, Bae HA, Huong NT, Lee SB (2008). Biochemical characterisation of a glycogen branching enzyme from *Streptococcus mutans*: Enzymatic modification of starch. Food Chem.

[CR36] Kittisuban P, Lee BH, Suphantharika M, Hamaker BR (2014). Slow glucose release property of enzyme-synthesized highly branched maltodextrins differs among starch sources. Carbohyd Polym.

[CR37] Le QT, Lee CK, Kim YW, Lee SJ, Zhang R, Withers SG, Kim YR, Auh JH, Park KH (2009). Amylolytically-resistant tapioca starch modified by combined treatment of branching enzyme and maltogenic amylase. Carbohyd Polym.

[CR38] Lee BH, Yan L, Phillips RJ, Reuhs BL, Jones K, Rose DR, Nichols BL, Quezada-Calvillo R, Yoo SH, Hamaker BR (2013). Enzyme-synthesized highly branched maltodextrins have slow glucose generation at the mucosal alpha-glucosidase level and are slowly digestible in vivo. PLoS ONE.

[CR39] Lee BH, Yan L, Phillips RJ, Reuhs BL, Jones K, Rose DR, Nichols BL, Quezada-Calvillo R, Yoo SH, Hamaker BR (2013). Enzyme-synthesized highly branched maltodextrins have slow glucose generation at the mucosal alpha-glucosidase level and are slowly digestible in vivo. PLoS ONE.

[CR40] Lee CK, Le QT, Kim YH, Shim JH, Lee SJ, Park JH, Lee KP, Song SH, Auh JH, Lee SJ, Park KH (2008). Enzymatic synthesis and properties of highly branched rice starch amylose and amylopectin cluster. J Agric Food Chem.

[CR41] Li C, Ban X, Zhang Y, Gu Z, Hong Y, Cheng L, Tang X, Li Z (2020). Rational Design of Disulfide Bonds for Enhancing the Thermostability of the 1,4-alpha-Glucan Branching Enzyme from *Geobacillus thermoglucosidans* STB02. J Agric Food Chem.

[CR42] Li L, Su L, Hu F, Chen S, Wu J (2020). Recombinant expression and characterization of the glycogen branching enzyme from *Vibrio vulnificus* and its application in starch modification. Int J Biol Macromol.

[CR43] Li WW, Li CM, Gu ZB, Qiu YJ, Cheng L, Hong Y, Li ZF (2016). Relationship between structure and retrogradation properties of corn starch treated with 1,4-alpha-glucan branching enzyme. Food Hydrocolloid.

[CR44] Li X, Miao M, Jiang H, Xue J, Jiang B, Zhang T, Gao Y, Jia Y (2014). Partial branching enzyme treatment increases the low glycaemic property and alpha-1,6 branching ratio of maize starch. Food Chem.

[CR45] Li Y, Ren J, Liu J, Sun L, Wang Y, Liu B, Li C, Li Z (2018). Modification by alpha-d-glucan branching enzyme lowers the in vitro digestibility of starch from different sources. Int J Biol Macromol.

[CR46] Lighezan L, Georgieva R, Neagu A (2016). The secondary structure and the thermal unfolding parameters of the S-layer protein from *Lactobacillus salivarius*. Eur Biophys J.

[CR47] Ludwig DS (2002). The glycemic index: physiological mechanisms relating to obesity, diabetes, and cardiovascular disease. JAMA.

[CR48] Man RC, Ismail AF, Ghazali NF, Fuzi SFZM, Illias RM (2015). Effects of the immobilization of recombinant *Escherichia coli* on cyclodextrin glucanotransferase (CGTase) excretion and cell viability. Biochem Eng J.

[CR49] Miao M, Jiang B, Cui SW, Zhang T, Jin Z (2015). Slowly digestible starch–a review. Crit Rev Food Sci Nutr.

[CR50] Mohtar NS, Abdul Rahman MB, Raja A, Rahman RN, Leow TC, Salleh AB, MatIsa MN (2016). Expression and characterization of thermostable glycogen branching enzyme from *Geobacillus mahadia* Geo-05. PeerJ.

[CR51] Na S, Park M, Jo I, Cha J, Ha NC (2017). Structural basis for the transglycosylase activity of a GH57-type glycogen branching enzyme from *Pyrococcus horikoshii*. Biochem Bioph Res Co.

[CR52] Pal K, Kumar S, Sharma S, Garg SK, Alam MS, Xu HE, Agrawal P, Swaminathan K (2010). Crystal Structure of Full-length *Mycobacterium tuberculosis* H37Rv Glycogen Branching enzyme insights of n-terminal beta-sandwich in substrate specificity and enzymatic activity. J Biol Chem.

[CR53] Pal K, Kumar S, Sharma S, Garg SK, Alam MS, Xu HE, Agrawal P, Swaminathan K (2010). Crystal structure of full-length *Mycobacterium tuberculosis* H37Rv glycogen branching enzyme: insights of N-terminal beta-sandwich in substrate specificity and enzymatic activity. J Biol Chem.

[CR54] Palomo M, Kralj S, van der Maarel MJ, Dijkhuizen L (2009). The unique branching patterns of Deinococcus glycogen branching enzymes are determined by their N-terminal domains. Appl Environ Microbiol.

[CR55] Palomo M, Pijning T, Booiman T, Dobruchowska JM, van der Vlist J, Kralj S, Planas A, Loos K, Kamerling JP, Dijkstra BW, van der Maarel MJEC, Dijkhuizen L, Leemhuis H (2011). *Thermus thermophilus* Glycoside Hydrolase Family 57 Branching enzyme crystal structure, mechanism of action, and products formed. J Biol Chem.

[CR56] Park I, Park M, Yoon N, Cha J (2019). Comparison of the structural properties and nutritional fraction of corn starch treated with thermophilic GH13 and GH57 alpha-Glucan Branching Enzymes. Foods.

[CR57] Peng H, Qian LM, Fu ZJ, Xin L, Hua Z, Woolf J, Xiao YZ, Gao Y (2021). Using a novel hyperthermophilic amylopullulanase to simplify resistant starch preparation from rice starches. J Funct Foods.

[CR58] Ping LF, Chen XY, Yuan XL, Zhang M, Chai YJ, Shan SD (2017). Application and comparison in biosynthesis and biodegradation by *Fusarium solani* and *Aspergillus fumigatus* cutinases. Int J Biol Macromol.

[CR59] Roussel X, Lancelon-Pin C, Vikso-Nielsen A, Rolland-Sabate A, Grimaud F, Potocki-Veronese G, Buleon A, Putaux JL (2013). Characterization of substrate and product specificity of the purified recombinant glycogen branching enzyme of *Rhodothermus obamensis*. Biochim Biophys Acta.

[CR60] Roussel X, Lancelon-Pin C, Vikso-Nielsen A, Rolland-Sabate A, Grimaud F, Potocki-Veronese G, Buleon A, Putaux JL (2013). Characterization of substrate and product specificity of the purified recombinant glycogen branching enzyme of *Rhodothermus obamensis*. Bba-Gen Subjects.

[CR61] Rumbak E, Rawlings DE, Lindsey GG, Woods DR (1991). Characterization of the *Butyrivibrio fibrisolvens* glgB gene, which encodes a glycogen-branching enzyme with starch-clearing activity. J Bacteriol.

[CR62] Santos CR, Tonoli CCC, Trindade DM, Betzel C, Takata H, Kuriki T, Kanai T, Imanaka T, Arni RK, Murakami MT (2011). Structural basis for branching-enzyme activity of glycoside hydrolase family 57: Structure and stability studies of a novel branching enzyme from the hyperthermophilic archaeon *Thermococcus Kodakaraensis* KOD1. Proteins-Structure Function and Bioinformatics.

[CR63] Sawada T, Itoh M, Nakamura Y (2018). Contributions of three starch branching enzyme isozymes to the fine structure of amylopectin in rice endosperm. Front Plant Sci.

[CR64] Shin HJ, Choi SJ, Park CS, Moon TW (2010). Preparation of starches with low glycaemic response using amylosucrase and their physicochemical properties. Carbohyd Polym.

[CR65] Suzuki E, Suzuki R (2016). Distribution of glucan-branching enzymes among prokaryotes. Cell Mol Life Sci.

[CR66] Takata H, Akiyama T, Kajiura H, Kakutani R, Furuyashiki T, Tomioka E, Kojima I, Kuriki T (2010). Application of branching enzyme in starch processing. Biocatal Biotransform.

[CR67] Takata H, Takaha T, Kuriki T, Okada S, Takagi M, Imanaka T (1994). Properties and active center of the thermostable branching enzyme from *Bacillus stearothermophilus*. Appl Environ Microbiol.

[CR68] Takata H, Takaha T, Okada S, Takagi M, Imanaka T (1996). Cyclization reaction catalyzed by branching enzyme. J Bacteriol.

[CR69] Tester RF, Karkalas J, Qi X (2004). Starch structure and digestibility enzyme-substrate relationship. World Poultry Sci J.

[CR70] Thiemann V, Saake B, Vollstedt A, Schafer T, Puls J, Bertoldo C, Freudl R, Antranikian G (2006). Heterologous expression and characterization of a novel branching enzyme from the thermoalkaliphilic anaerobic bacterium *Anaerobranca gottschalkii*. Appl Microbiol Biotechnol.

[CR71] van der Maarel MJ, van der Veen B, Uitdehaag JC, Leemhuis H, Dijkhuizen L (2002). Properties and applications of starch-converting enzymes of the alpha-amylase family. J Biotechnol.

[CR72] Van der Maarel MJEC, Ter Veer BCA, Vrieling-Smit A, Delnoye DAP (2014) Methods and means for coating paper by film coating. WO2014003556A1

[CR73] Van der Maarel MJEC, Vos A, Sanders P, Dijkhuizen L (2003). Properties of the glucan branching enzyme of the hyperthermophilic bacterium *Aquifex aeolicus*. Biocatal Biotransform.

[CR74] Wang Z, Xin C, Li C, Gu Z, Cheng L, Hong Y, Ban X, Li Z (2019). Expression and characterization of an extremely thermophilic 1,4-alpha-glucan branching enzyme from *Rhodothermus obamensis* STB05. Protein Expr Purif.

[CR75] Wickramasinghe HAM, Blennow A, Noda T (2009). Physico-chemical and degradative properties of in-planta re-structured potato starch. Carbohyd Polym.

[CR76] Wu S, Liu Y, Yan Q, Jiang Z (2014). Gene cloning, functional expression and characterisation of a novel glycogen branching enzyme from *Rhizomucor miehei* and its application in wheat breadmaking. Food Chem.

[CR77] Xia C, Zhong L, Wang J, Zhang L, Chen X, Ji H, Ma S, Dong W, Ye X, Huang Y, Li Z, Cui Z (2021). Structural and digestion properties of potato starch modified using an efficient starch branching enzyme AqGBE. Int J Biol Macromol.

[CR78] Xiao Y, Shen M, Luo Y, Ren Y, Han X, Xie J (2020). Effect of Mesona chinensis polysaccharide on the pasting, rheological, and structural properties of tapioca starch varying in gelatinization temperatures. Int J Biol Macromol.

[CR79] Yang G, Yang D, Wang X, Cao W (2021). A novel thermostable cellulase-producing *Bacillus licheniformis* A5 acts synergistically with *Bacillus subtilis* B2 to improve degradation of Chinese distillers' grains. Bioresour Technol.

[CR80] Yang S, Qiaojuan Y, Jiang Z, Fan G, Wang L (2008). Biochemical characterization of a novel thermostable beta-1,3–1,4-glucanase (lichenase) from *Paecilomyces thermophila*. J Agric Food Chem.

[CR81] Ye X, Liu W, Ma S, Chen X, Qiao Y, Zhao Y, Fan Q, Li X, Dong C, Fang X, Huan M, Han J, Huang Y, Cui Z, Li Z (2021). Expression and characterization of 1,4-alpha-glucan branching enzyme from Microvirga sp MC18 and its application in the preparation of slowly digestible starch. Protein Expr Purif.

[CR82] Zeeman SC, Kossmann J, Smith AM (2010). Starch: its metabolism, evolution, and biotechnological modification in plants. Annu Rev Plant Biol.

[CR83] Zhang X, Leemhuis H, van der Maarel M (2019). Synthesis of highly branched alpha-glucans with different structures using GH13 and GH57 glycogen branching enzymes. Carbohydr Polym.

[CR84] Zhang Z, Yang J, Xie P, Gao Y, Bai J, Zhang C, Liu L, Wang Q, Gao X (2020). Characterization of a thermostable phytase from *Bacillus licheniformis* WHU and further stabilization of the enzyme through disulfide bond engineering. Enzyme Microb Technol.

[CR85] Zhou W, Zhao S, He S, Ma Q, Lu X, Hao X, Wang H, Yang J, Zhang P (2020). Production of very-high-amylose cassava by post-transcriptional silencing of branching enzyme genes. J Integr Plant Biol.

