# Supplementary material for: Biochemical characteristics and potential application of a thermostable starch branching enzyme from Bacillus licheniformis
Source: AMB Express. 2023 Jan 20;13:8. doi: 10.1186/s13568-023-01511-4 (PMC9859979; doi:10.1186/s13568-023-01511-4)
Supplement: Supplementary file 1 — Additional file 1: Table S1. Strains and plasmids used in this study. Table S2. The identity percentage for alignment between bl-GBE and ten GBE sequences obtained from literature mining. Table S3. Relevant index of protein purification. [file 13568_2023_1511_MOESM1_ESM.docx]

**AMB Express**

**Biochemical characteristics and potential application of a thermostable starch branching enzyme from *Bacillus licheniformis***

# Ting Yang^1^, Qianyu Hu^1^, Yu Liu^1^, Rui Xu^1^, Dongrui Wang^1^, Zhongyi Chang^1^, Mingfei Jin^1*,^ Jing Huang^1*^

^1^ School of Life Sciences, East China Normal University, Shanghai 200241, China. * Correspondence authors.

**Mingfei Jin**

[mfjin@bio.ecnu.edu.cn](mailto:mfjin@bio.ecnu.edu.cn).

**Jing Huang**

[jhuang@bio.ecnu.edu.cn](mailto:jhuang@bio.ecnu.edu.cn).

**Table S1** Strains and plasmids used in this study

| **Strain/Plasmid** | **Description** | | **Source** | |
| --- | --- | --- | --- | --- |
| **Strain** |  |  | |  |
| *E. coli* DH5α | Host for plasmid replication | Sangon Biotech (Shanghai, China) | |  |
| *E. coli* BL21(DE3) | Host for protein expression | Merck (Darmstadt, Germany) | |  |
| *Bacillus licheniformis* ATCC14580 | Host for Source of gbe gene | Merck (Darmstadt, Germany) | |  |
| **Plasmid** |  |  | |  |
| pET32a (+)-gbe | The plasmid pET32a (+) containing gbe | This study | |  |

**Table S2** The identity percentage for alignment between bl-GBE and ten GBE sequences obtained from literature mining.

| **Organism** | **UniProt Accession** | **Identity (%** | **reference** |
| --- | --- | --- | --- |
| *Bacillus stearothermophilus* | BAA19588.1 | 64.2 | (Choi et al. 2009) |
| *Aquifex aeolicus* VF5 | AAC06895.1 | 49.8 | (Van Der Maarel et al. 2003) |
| *Rhodothermus marinus* | AEN72909.1 | 47.1 | (Yoon et al. 2008) |
| *Anaerobranca gottschalkii* | CAJ38414.1 | 52.6 | (Thiemann et al. 2006) |
| *Bacillus cereus* | AIY74141.1 | 62.2 | (Takata et al. 2010) |
| *Deinococcus radiodurans* | QIP29803.1 | 44.0 | (Palomo et al. 2009) |
| *Deinococcus geothermalis* | WP_041221078.1 | 45.1 | (Palomo et al. 2009) |
| *Escherichia coli* | CUU95581.1 | 44.9 | (Guan et al. 1997) |
| *Mycobacterium tuberculosis* | OHO20383.1 | 43.4 | (Garg et al. 2007; Pal et al. 2010) |
| *Streptococcus mutans* | QFG44887.1 | 44.0 | (Kim et al. 2008) |

**Table S3** Relevant index of protein purification.

| **Step** | **Enzyme activity (U/mg)** | **Total protein(mg)** | **Protein recovery rate (%)** |
| --- | --- | --- | --- |
| Denaturation | / | 143.2 | / |
| Renaturation | 60.8 | 56.8 | 39.7 |
| Ni-NTA column | 77.2 | 33.4 | 23.3 |

**References**

Choi SS, Danielewska-Nikiel B, Kojima I, Takata H (2009) Safety evaluation of 1,4-alpha-glucan branching enzymes from *Bacillus stearothermophilus* and *Aquifex aeolicus* expressed in *Bacillus subtilis*. Food Chem Toxicol 47(8):2044-51. <http://doi.org/10.1016/j.fct.2009.05.019>

Garg SK, Alam MS, Kishan KV, Agrawal P (2007) Expression and characterization of alpha-(1,4)-glucan branching enzyme Rv1326c of *Mycobacterium tuberculosis* H37Rv. Protein Expr Purif 51(2):198-208. <http://doi.org/10.1016/j.pep.2006.08.005>

Guan H, Li P, Imparl-Radosevich J, Preiss J, Keeling P (1997) Comparing the properties of *Escherichia coli* branching enzyme and maize branching enzyme. Arch Biochem Biophys 342(1):92-8. <http://doi.org/10.1006/abbi.1997.0115>

Kim EJ, Ryu SI, Bae HA, Huong NT, Lee SB (2008) Biochemical characterisation of a glycogen branching enzyme from *Streptococcus mutans*: Enzymatic modification of starch. Food Chem 110(4):979-84. <http://doi.org/10.1016/j.foodchem.2008.03.025>

Pal K, Kumar S, Sharma S, Garg SK, Alam MS, Xu HE, Agrawal P, Swaminathan K (2010) Crystal structure of full-length *Mycobacterium tuberculosis* H37Rv glycogen branching enzyme: insights of N-terminal beta-sandwich in substrate specificity and enzymatic activity. J Biol Chem 285(27):20897-903. <http://doi.org/10.1074/jbc.M110.121707>

Palomo M, Kralj S, van der Maarel MJ, Dijkhuizen L (2009) The unique branching patterns of *Deinococcus* glycogen branching enzymes are determined by their N-terminal domains. Appl Environ Microbiol 75(5):1355-62. <http://doi.org/10.1128/AEM.02141-08>

Takata H, Akiyama T, Kajiura H, Kakutani R, Furuyashiki T, Tomioka E, Kojima I, Kuriki T (2010) Application of branching enzyme in starch processing. Biocatalysis and Biotransformation 28(1):60-63.

Thiemann V, Saake B, Vollstedt A, Schafer T, Puls J, Bertoldo C, Freudl R, Antranikian G (2006) Heterologous expression and characterization of a novel branching enzyme from the thermoalkaliphilic anaerobic bacterium *Anaerobranca gottschalkii*. Appl Microbiol Biotechnol 72(1):60-71. <http://doi.org/10.1007/s00253-005-0248-7>

Van Der Maarel M, Vos A, Sanders P, Dijkhuizen L (2003) Properties of the glucan branching enzyme of the hyperthermophilic bacterium *Aquifex aeolicus*. Biocatalysis and Biotransformation 21(4-5):199-207.

Yoon SA, Ryu SI, Lee SB, Moon TW (2008) Purification and characterization of branching specificity of a novel extracellular amylolytic enzyme from marine hyperthermophilic *Rhodothermus marinus*. J Microbiol Biotechnol 18(3):457-64.
